# The inequality in the burden of road traffic injuries among children aged 0-14 years in Iran: estimates from the global burden of disease study 2019

**DOI:** 10.1186/s12889-025-25186-7

**Published:** 2025-11-14

**Authors:** Elham Goodarzi, Zaher Khazaei, Ali Karamoozian, Farzaneh AsadiLari, Ahmad Naghibzadeh-Tahami

**Affiliations:** 1 Social Determinants of Health Research Center, Lorestan University of Medical Sciences, Khorramabad, Iran; 2 Social Determinants of Health Research Center, Iran University of Medical Sciences, Tehran, Iran; 3 Non-Communicable Diseases Research Center, Sabzevar University of Medical Sciences, Sabzevar, Iran; 4 Department of Biostatistics and Epidemiology, Faculty of Health, Kerman University of Medical Sciences, Kerman, Iran; 5 Medical Informatics Research Center, Institute for Futures Studies in Health, Kerman University of Medical Sciences, Kerman, Iran; 6 HIV/STI Surveillance Research Center, and WHO Collaborating Center for HIV Surveillance, Institute for Futures Studies in Health, Kerman University of Medical Sciences, Kerman, Iran; 7 Health Foresight and Innovation Research Center, Institute for Futures Studies in Health, Kerman University of Medical Sciences, Kerman, Iran

**Keywords:** Road injuries, Children, HDI, Global burden of disease, Iran

## Abstract

**Background:**

Road traffic injuries (RTIs) remain a leading cause of death and disability, posing a significant public health challenge. This study analyzes the relationship between the Human Development Index (HDI) and epidemiological patterns of RTIs among children aged 0–14 years. The primary objective is to identify and examine inequalities in the burden of these injuries as a major contributor to mortality and disability in this age group.

**Methods:**

The current study is a descriptive-analytical study and children aged 0–14 in Iran are the target population of the study. The data regarding incidence, mortality, and years of healthy life lost due to disability (YLD), years of life lost due to premature mortality (YLL), and disability-adjusted life years (DALY) caused by road traffic injuries was collected from Global Burden of Disease 2019 (GBD) from 2010 to 2019. In order to evaluate the Pearson correlation between these indexes with the human development index (HDI), a correlation test was used. All statistical analyses were performed using Stata version 17 (StataCorp LLC).

**Results:**

The result also showed that in 2019 the most cause of death 3.86(3.03, 4.94) years of life lost due to premature mortality 314.9(246.9, 400.3), and DALY 317.4(249.9, 402.42) was caused by motor vehicle injuries and the most of the incidences 190.8(114.7, 285.6) and DALY 3.12(1.96, 4.56) in 2019 was caused by Cyclist Road injuries. A strong correlation has been seen among road traffic mortality (*r* = -0.613, *p*-value = 0.0002), DALY rate (*r* = -0.622, *p*-value = 0.0002), and YLL (*r* = -0.622, *p*-value = 0.0002) with HDI.

**Conclusion:**

Although there has been a decrease in road traffic mortality in children, still some road safety actions must be taken to reduce road injuries specifically in provinces with lower HDI.

## Introduction

Road traffic injury (RTIs) is still a global epidemic and due to its high mortality rate on the international scale, it is a public health priority [1]. The death toll has been updated from 1.35 million to 1.19 million. This reflects the most recent comprehensive estimate from the WHO [2]. Based on the GBD, currently, road injuries are the 7th most important reason for DALY which show the main economic and social burden [3]. Road safety is explicitly targeted under the Sustainable Development Goals (SDGs). Target 3.6 aims to halve the number of global deaths and injuries from RTIs by 2030, using 2015 as the baseline year [4].

Compared to countries such as the United States of America in which the average road traffic mortality has dropped over time, in other parts of the world, such as some regions of Asia, the average road traffic mortality has either remained steady or has gone up [5]. Specifically speaking, in some parts of the western Pacific Ocean and Southeast Asia, the average rate of road traffic mortalities is higher than 18.5 per 100,000 persons compared with 16.1 per 100,000 persons in the United States of America [6]. More than 90% of road traffic injuries happen in low or average-income countries and pedestrians, cyclists, and motorcyclists are the vulnerable ones who comprise half of such road traffic injuries [3].

In Iran, the age-standardized mortality rate caused by incidence was respectively 59–87 and 34–82 per 100,000 persons in 1990 and 2016 [7] and road traffic injuries are the second most leading cause of DALY in Iran [7].

Although road traffic injuries impact all age groups, the impact of such incidence in the young age group of society is felt more. The estimate of the GBD indicates that road traffic injuries are the main cause of death in children age groups 0–14 [8]. Since there is more risk for road traffic injuries in urban areas and a huge rate of such incidence takes place in urban areas, children and adolescents, in low and average-income countries, are an important group that is more vulnerable to road injuries [8].

Different studies from different countries have shown the correlation between HDI and mortality rate from road accidents. Studies that were conducted in developing countries from 2009 to 2018 confirmed that there was no correlation between HDI and road safety, however, in developed counties where there are more opportunities to invest in infrastructure, education, and health care systems, the mortality rate from road accidents is lower. Since there is a significant road transportation dependency in most regions, to effectively reduce the burden of road injuries, road safety must be improved [9]. Providing accurate and authentic information to policymakers and healthcare planners is a must for it informs them to allocate sources where they are most needed and also helps them to focus their attention in more targeted areas [10].

Therefore, if resources are to be allocated to intervention and safety policies, policymakers need to have a clear understanding of the problems. So it is from this perspective that we’ve performed this study to investigate the incidence, mortality, and burden of road accidents in children aged 0–14 and their correlation with the human development index.

## Methodology

This study employed an ecological correlational design to assess the relationship between socioeconomic development and the burden of RTIs in Iran. We analyzed longitudinal (time-series) data at the province level for the period 2010–2019.Data on incidence, mortality, YLD, YLL, and DALY due to RTIs in children aged 0 to 14 years in Iran from 2010 to 2019 by province were extracted in Excel files(CSV) from http://ghdx.healthdata.org/gbd-results-tool. GBD 2019 has calculated the mortality rate, YLL, YLD, and DALY in 23 age groups among men and women in 204 countries including Iran from 1990 to 2019 [11]. GBD has followed a systematic approach to provide enough evidence in regard to health consequences in humans. The data used by GBD have been collected from various number of sources. The framework for an accurate estimation by GBD 2019 has been discussed before [12–14]. The data for estimating Iran traffic road injuries have been collected from different sources including the national death registration system, the national system of forensic medicine, hospitals, and Iran’s demographic & health surveys [15, 16].

### Definition

#### Age groups

This study focused on the burden of RTIs in children and young adolescents aged 0 to 14 years. This age group was selected a priori due to its specific vulnerability and public health importance.

#### Composite metrics: DALY, YLD and YLL

To estimate DALY, the rate of YLD and YLL have been added together. DALY is the years of life lost due to accidents and was computed by the difference between the current state of healthcare and the ideal state that any person of any age could spend his time in that ideal state. Years of life lost due to road traffic injuries were computed by (YLL). By multiplying YLL by the number of death in each age group, GBD computed the average life expectancy of that group. Disability caused by road injuries was computed by the years of healthy life lost due to disability (YLD). YLD is the result of a computation calculated by the prevalence of a health outcome multiplied by its disability weight [10, 17]. In the current study, point estimations as well as Ul 95% were used for all the indexes [12].

#### Road user categories

The Global Burden of Disease (GBD) study classified and defined RTIs according to the International Classification of Diseases, Tenth Revision (ICD-10). Pedestrian: Injuries involving a pedestrian injured in a transport accident (ICD-10 codes V01-V09). Cyclist: Injuries involving a pedal cyclist injured in a transport accident (ICD-10 codes V10-V19). Motorcyclist: Injuries involving a motorcycle rider or passenger injured in a transport accident (ICD-10 codes V20-V29). Motor vehicle occupant: Injuries involving an occupant of a four-wheeled motor vehicle injured in a transport accident (ICD-10 codes V30-V89, encompassing cars, trucks, buses, and other motor vehicles). Other road user: This category includes injuries to unspecified road users and those not covered in the above categories (e.g., injuries involving other specified transport accidents, V99) [2, 9].

#### Human Development Index (HDI)

The HDI, published by the United Nations Development Programme (UNDP), is a key summary measure of average achievement in three fundamental dimensions of human development: a long and healthy life (measured by life expectancy at birth), access to knowledge (measured by mean and expected years of schooling), and a decent standard of living (measured by Gross National Income per capita). The HDI is a composite statistic represented as a unitless value ranging from 0 to 1, with higher values indicating higher levels of development. For the purpose of this study, subnational HDI data for the year 2019 were obtained for all provinces of Iran from the https://globaldatalab.org/shdi/pop/IRN/?levels=1%2B4&interpolation=1&extrapolation=0&nearest_real=0.

The year 2019 was selected to ensure temporal alignment with the endpoint of our outcome data series (2010–2019) and to represent the most recent pre-pandemic developmental context [18–20].

### Statistical analysis

The extracted data comprised annual, province-level estimates for the period 2010–2019. All analyses were based on the age-standardized rates (mortality, incidence, DALY, YLL and YLD) provided in the GBD 2019 dataset. These rates are expressed per 100,000 population. These rates, which are essential for enabling unbiased comparisons across provinces with differing age structures, were calculated by the GBD collaborative group using the GBD World Standard Population as the reference. The GBD's internal demographic modeling ensures the population denominators used for rate calculations are consistent and reliable across all locations and years. We utilized these pre-calculated rates directly without further adjustment.

To assess the ecological association between the burden of RTIs and the HDI, we employed Pearson correlation analysis. The strength and direction of the linear relationship were visualized using scatter plots. All statistical analyses were performed using Stata version 17 (StataCorp LLC), and a two-sided p-value of less than 0.05 was considered statistically significant.

## Result

**Fig. 1:**
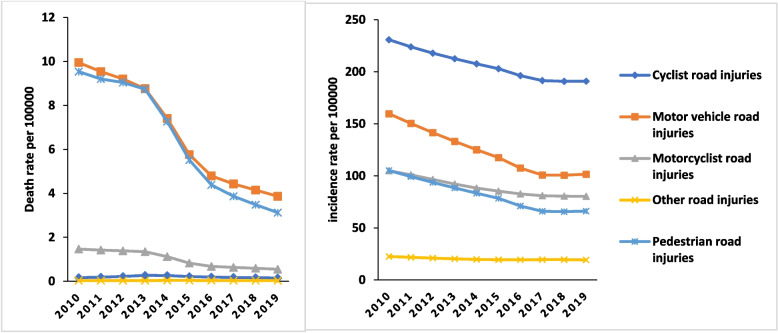
Trend incidence rate and death rate of RTIs per 100000 Subcategories in children 0–14 year in Iran during 2010–2019. (Source: Global Burden of Disease 2019)

Figure 1 shows the mortality and incidence rate (per 100,000 persons) in RTIs in children aged 0–14 from 2010 to 2019. Based on road injuries in children 0–14, there has been a downward trend and compared to 2010 there has been a decrease in mortality rate in 2019.

Table 1 shows the mortality incidence rate and burden of RTIs in children from 2010 in comparison with 2019 and the difference between these two years. As it is seen, most of the RTIs in 2010 and 2019 is caused by cyclist road injuries.

**Table 1:** Incidence, Death, YLL, YLD and DALY Rates by RTIs Subcategories and APC in Iran, 2010 and 2019, Both sex. (Source: Global Burden of Disease 2019)

Subcategory		Rate per 100000 in 0–14 year (UI 95%)		APC * 2010–2019
		**2010**	**2019**	
Road injuries	Incidence	623.05 (445.4, 868.6)	458.10 (321.4, 663.7)	−0.26 (−0.31, −0.21)
	Death	21.14 (18.6, 24.11)	7.72 (6.19, 9.42)	−0.63 (−0.7, −0.56)
	YLL	1736.69 (1527.07, 1986.9)	626.85 (500.63, 764.8)	−0.63 (−0.71, −0.56)
	YLD	15.51 (10.5, 21.64)	10.60 (7.16, 14.81)	−0.31 (−0.34, −0.28)
	DALY	1752.2 (1543.6, 2001.2)	637.45 (511.3, 774.52)	−0.63 (−0.7, −0.55)
Pedestrian road injuries	Incidence	105.10 (62.2, 170.15)	66.1 (37.05, 111.8)	−0.37 (−0.43, −0.31)
	Death	9.53 (8.21, 11.29)	3.12 (2.35, 3.86)	−0.67 (−0.74, −0.59)
	YLL	782.3 (671.9, 930.2)	253.3 (189.6, 314.6)	−0.67 (−0.75, −0.59)
	YLD	3.6 (2.29, 5.23)	2.08 (1.3, 3.08)	−0.42 (−0.45, −0.38)
	DALY	785.9 (675.9, 933.9)	255.40 (191.7, 316.9)	−0.67 (−0.75, −0.59)
Cyclist road injuries	Incidence	230.78 (141.2, 339.79)	190.80 (114.7, 285.6)	−0.17 (−0.23, −0.1)
	Death	0.16 (0.13, 0.24)	0.15 (0.09, 0.25)	−0.08 (−0.7, 0.22)
	YLL	13.31 (10.69, 19.22)	12.08 (7.74, 20.35)	−0.09 (−0.37, 0.21)
	YLD	3.90 (2.51, 5.71)	3.12 (1.96, 4.56)	−0.21 (−0.25, −0.17)
	DALY	17.30 (14.04, 23.64)	15.21 (10.59, 23.38)	−0.12 (−0.33, 0.13)
Motorcyclist road injuries	Incidence	104.9 (63.8, 161.7)	80.28 (47.6, 128.6)	−0.23 (−0.3, −0.16)
	Death	1.46 (1.19, 2.14)	0.54 (0.35, 0.88)	−0.62 (−0.73, −0.47)
	YLL	118.31 (96.04, 173.57)	43.98 (28.65, 72.01)	−062 (−0.73, −0.48)
	YLD	3.40 (2.3, 4.98)	2.58 (1.71, 3.72)	−0.25 (−0.29, −0.22)
	DALY	121.80 (99.74, 177.42)	46.57 (31.43, 74.6)	−0.61 (−0.72, −0.47)
Motor vehicle road injuries	Incidence	159.6 (97.3, 251.5)	101.60 (58.57, 170.1)	−0.36 (−0.42, −0.3)
	Death	9.94 (8.34, 11.57)	3.86 (3.03, 4.94)	−0.61 (−0.7, −0.48)
	YLL	819.80 (682.92, 957.35)	314.90 (246.9, 400.3)	−0.61 (−0.71, −0.48)
	YLD	4.04 (2.64, 6)	2.48 (1.5, 3.78)	−0.38 (−0.42, −0.34)
	DALY	823.90 (685.7, 961.3)	317.40 (249.9, 402.42)	−0.61 (−0.7, −0.48)
Other road injuries	Incidence	22.51 (11.4, 42.49)	19.25 (9.63, 37.95)	−0.14 (−0.21, −0.07)
	Death	0.03 (0.02, 0.04)	0.03 (0.01, 0.04)	−0.11 (−0.45, 0.3)
	YLL	2.82 (2.26, 3.96)	2.48 (1.45, 3.98)	−0.12 (−0.45, 0.3)
	YLD	0.36 (0.21, 0.55)	0.30 (0.18, 0.47)	−0.15 (−0.19, −0.11)
	DALY	3.19 (2.59, 4.32)	2.70 (1.74, 4.26)	−0.12 (−0.43, 0.24)

^*^APC Annual Percentage Change

In 2010, the highest death rate 9.94(8.34, 11.57) and in 2019, 3.86(3.03, 4.94) was caused by motor vehicle injuries. In 2010, the most years of life lost due to premature mortality 819.8(682.92, 957.35) and in 2019, 314.9(246.9, 400.3) (per 100,000 persons) were caused by motor vehicle injuries.

In 2010, the most years of life lost due to disability 4.04(2.64, 6) was caused by motor vehicle injuries, and in 2019, the most years of life lost due to disability 3.12(1.96, 4.56) was caused by cyclist road injuries. The most DALY reported in 2010, 823.9(685.7, 961.3) and in 2019, 317.4(249.9, 402. 42) were caused by motor vehicle injuries as well. The change rate for all the road injury subcategories has been negative, showing that in 2019 there has been a decrease compared with 2010.

Table 2 shows RTIs in children 0–14 in terms of gender. Most of the road injuries in boys in 2010–2019 were related to cyclist road injuries which compared to 2010 in 2019 some changes have been seen −0.18(−0.24, −0.11).

**Table 2:** Incidence, Death, YLL, YLD and DALY Rates by RTIs Subcategories and APC in Iran, 2010 and 2019 By Sex. (Source: Global Burden of Disease 2019)

Subcategory		Male			Female		
		2010	2019	APC* 2010–2019 (UI 95%)	2010	2019	APC* 2010–2019 (UI 95%)
Road injuries	Incidence	698.20	511.81	−0.26 (−0.31, −0.21)	544.29	401.65	−0.26 (−0.31, −0.21)
	Death	25.24	9.41	−0.62 (−0.7, −0.55)	16.85	5.936	−0.64 (−0.72, −0.56)
	YLL	2063.56	761.40	−0.63 (−0.7, −0.55)	1394.13	485.17	−0.65 (−0.72, −0.57)
	YLD	17.27	11.86	−0.31 (−0.34, −0.27)	13.65	9.26	−0.32 (−0.35, −0.28)
	DALY	2080.84	773.26	−0.62 (−0.7, −0.55)	1407.79	494.44	−0.64 (−0.72, −0.56)
Pedestrian road injuries	Incidence	114.93	73.81	−0.35 (−0.42, −0.29)	94.80	57.98	−0.38 (−0.45, −0.33
	Death	11.36	4.089	−0.64 (−0.72, −0.51)	7.61	2.10	−0.72 (−0.8, −0.6)
	YLL	928.62	331.02	−0.64 (−0.73, −0.51)	629.03	171.49	−0.72 (−0.83, −0.61)
	YLD	3.80	2.27	−0.4 (−0.44, −0.35)	3.40	1.89	−0.44 (−0.48,−0.4)
	DALY	932.42	333.29	−0.64 (−0.73, −0.51)	632.43	173.39	−0.72 (−0.82, −0.61)
Cyclist road injuries	Incidence	268.09	218.58	−0.18 (−0.24, −0.11)	191.68	161.73	−0.15 (−0.22, −0.08)
	Death	0.26	0.22	−0.15 (−0.51, 0.21)	0.06	0.08	0.17 (−0.14, 0.78)
	YLL	20.74	17.47	−0.15 (−0.51, 0.21)	5.51	6.41	0.16 (−0.14, 0.78)
	YLD	4.771	3.68	−0.22 (−0.27, −0.18)	3.16	2.53	−0.19 (−0.24, −0.15)
	DALY	25.51	21.16	−0.17 (−0.46, 0.14)	8.68	8.95	0.03 (−0.16, 0.41)
Motorcyclist road injuries	Incidence	123.22	93.27	−0.24 (−0.31, −0.17)	85.79	66.61	−0.22 (−0.3, −0.14)
	Death	2.02	0.719	−0.64 (−0.77,−0.48)	0.88	0.37	−0.57 (−0.72, −0.28)
	YLL	162.04	57.04	−0.64 (−0.77, −0.49)	72.48	30.23	−0.58 (−0.73, −0.28)
	YLD	3.99	2.96	−0.25 (−0.29, −0.21)	2.97	2.19	−0.26 (−0.3, −0.22)
	DALY	166.04	60.01	−0.63 (−0.76, −0.48)	75.45	32.43	−0.57 (−0.72, −0.28)
Motor vehicle road injuries	Incidence	165.20	103.49	−0.37 (−0.43, −0.31)	153.93	99.63	−0.35 (−0.41, −0.32)
	Death	11.56	4.34	−0.62 (−0.75, −0.47)	8.25	3.361	−0.59 (−0.67, −0.37)
	YLL	949.04	352.57	−0.62 (−0.75,−0.47)	684.54	275.38	−0.59 (−0.68, −0.37)
	YLD	4.272	2.58	−0.39 (−0.43, −0.34)	3.81	2.38	−0.37 (−0.41, −0.32)
	DALY	953.31	355.16	−0.62 (−0.75, −0.47)	688.35	277.77	−0.59 (−0.67, −0.37)
Other road injuries	Incidence	26.75	22.64	−0.15 (−0.22, −0.08)	18.073	15.68	−0.13 (−0.2, −0.05)
	Death	0.037	0.04	0.06 (−0.38, 0.64)	0.03	0.02	−0.35 (−0.59, −0.04)
	YLL	3.092	3.27	0.06 (−0.39, 0.64)	2.55	1.64	−0.35 (−0.6, −0.04)
	YLD	0.432	0.35	−0.16 (−0.21,−0.12)	0.29	0.25	−0.14 (−0.18, −0.09)
	DALY	3.52	3.63	0.03 (−0.36, 0.53)	2.84	1.89	−0.33 (−0.55, −0.05)

^*^APC Annual Percentage Change

Most of the road injuries in boys and girls in 2010 and 2019 were caused by motor vehicle injuries. The rate of change in 2019 compared with 2010 in boys was −0.62(−0.75, −0.47), and in girls was −0.599(−0.68, −0.37) which shows that there has been a decrease in both groups.

In 2010 and 2019, the most years of life lost due to premature mortality in boys and girls was caused by motor vehicle injuries. The rate change in 2019 for boys was −0.62(−0.75, −0.47), and for girls, it was −0.59(−0.68, −0.37), compared with 2010, the rate has decreased in both groups.

In 2010 and 2019 the most years of life lost due to disability in boys was caused by cyclist road injuries, the rate changes in 2019 were −0.22(−0.27, −0.18) which shows that there has been a decrease compared with 2010.

In 2010 and 2019 the most years of life lost due to disability in girls was caused by motor vehicle injuries, the rate changes in 2019 were −0.37(−0.41, −0.32) which shows that there has been a decrease compared with 2010.

In 2010 and 2019, most DALY rates of road accidents in boys and girls were caused by motor vehicle injuries and the rate of changes in 2019 for both groups was negative compared to 2010, the rate change for boys was −0.62(−0.75, −0.47) and for girls, it was −0.59(−0.67, −0.37).

For other road injuries in boys, the rate of mortality change was 0.06(−0.38, 0.64) and the rate of change for years of life lost due to premature mortality in 2010 was 0.06(−0.39, 0.64) which shows that the rate was positive and higher compared with 2019.

**Fig. 2:**
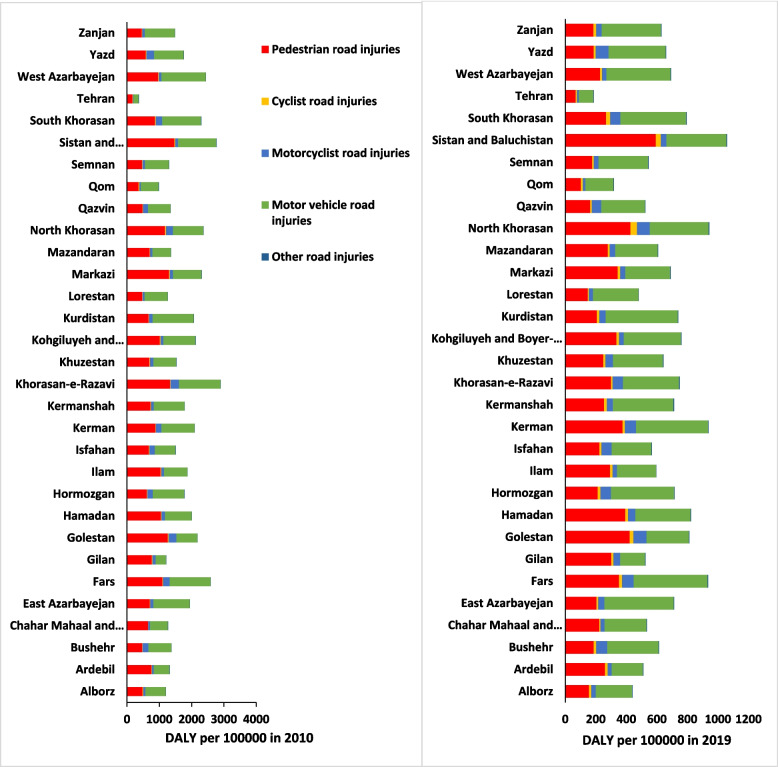
RTIs DALY Rates per 100,000 Persons by State, Iran 2019 and 201. (Source: Global Burden of Disease 2019)

Figure 2 shows the DALY rate of road accidents in 2010 and compares it with 2019 by provinces. As it is seen in 2010, the most DALY in all the provinces is caused by pedestrian road injuries, and Sistan and Baluchestan province with a rate of 1470.23 per 100,000 persons has the highest rate, and Tehran with a rate of 168.98 per 100,000 persons has the lowest rate. In 2019 in all the provinces except Sistan and Baluchestan, North Khorasan, Markazi, Hamadan, Gilan, Golestan, and Ardebil most of the burden of road traffic injuries was caused by pedestrian road injuries. The highest burden of motor vehicle injuries was related to Fars province with a rate of (482.93 per 100,000 persons) and the lowest was related to Tehran province with a rate of (94.22 per 100,000 persons).

**Fig. 3:**
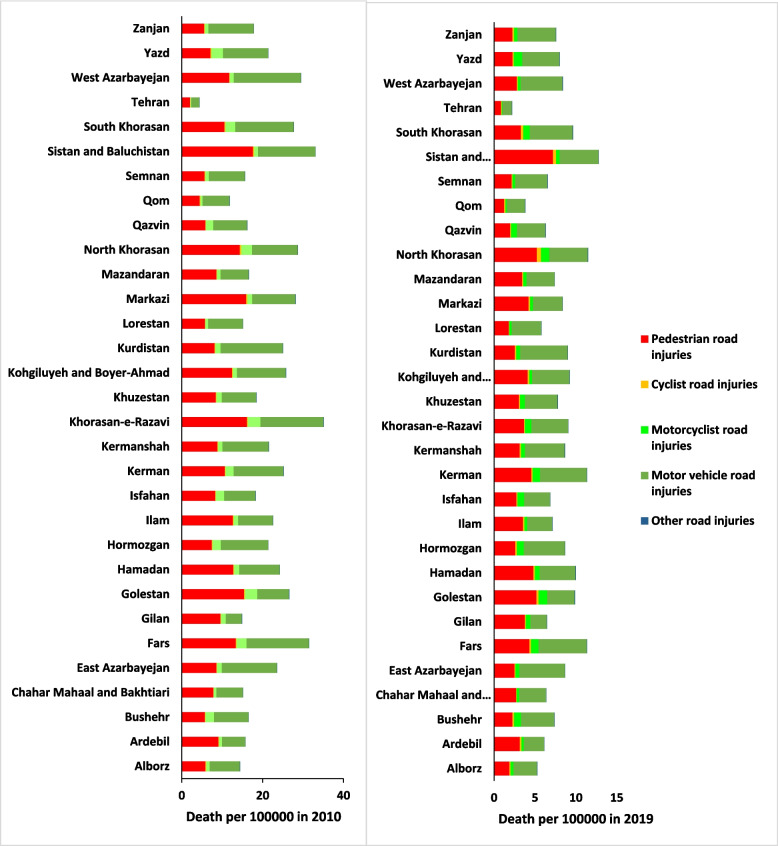
RTIs Death Rates per 100,000 Persons by State, Iran 2019 and 2010. (Source: Global Burden of Disease 2019)

The finding indicates that in 2010 the highest death rate in provinces such as North Khorasan, Markazi, Mazandaran, Kohgiluyeh and Boyer-Ahmad, Razavi Khorasan, Ilam, Hamadan, Golestan, Chaharmahal and Bakhtiari and Ardebil was caused by pedestrian road injuries and in rest of the provinces, the highest death rate was caused by motor vehicle injuries. In 2019, the highest death rate in provinces such as Sistan and Baluchestan, North Khorasan, Mazandaran, Markazi, Ilam, Hamadan, Golestan, Gilan, and Ardebil was caused by pedestrian road injuries, and in the rest of the provinces, the highest death rate was caused by motor vehicle injuries (Fig. 3).

**Fig. 4:**
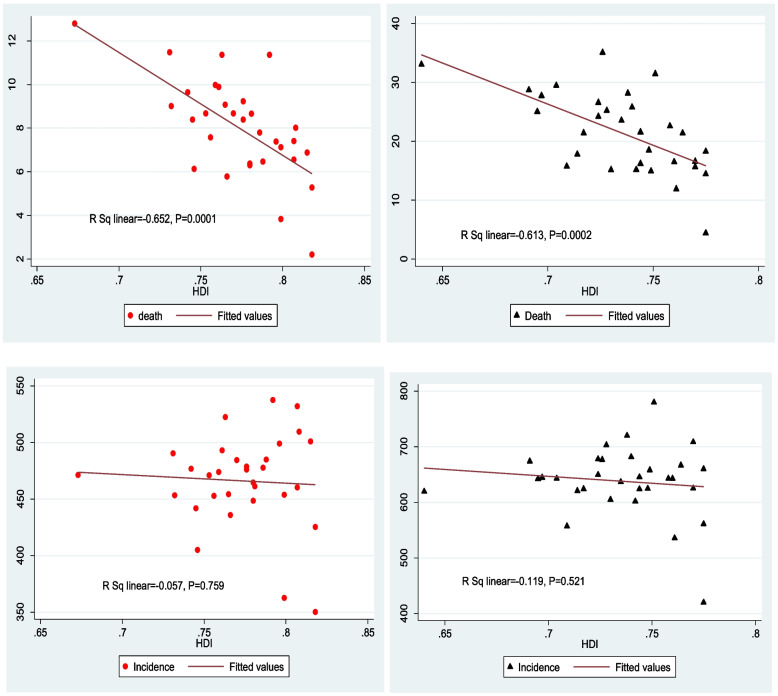
Correlation between the HDI with incidence and Death rates of RTIs in Iran in 2010 and 2019

The finding showed that there was a strong negative correlation between the road injury death rate and the human development index in 2010 (*r* = −0662, *p*-value = 0.0001) and in 2019 (*r* = −0.613, *p*-value = 0.0002). The finding also showed that there was a negative correlation between the incidence rate and HDI in 2010 and 2019, however, statistically speaking, it wasn’t a significant correlation (Fig. 4).

**Fig. 5:**
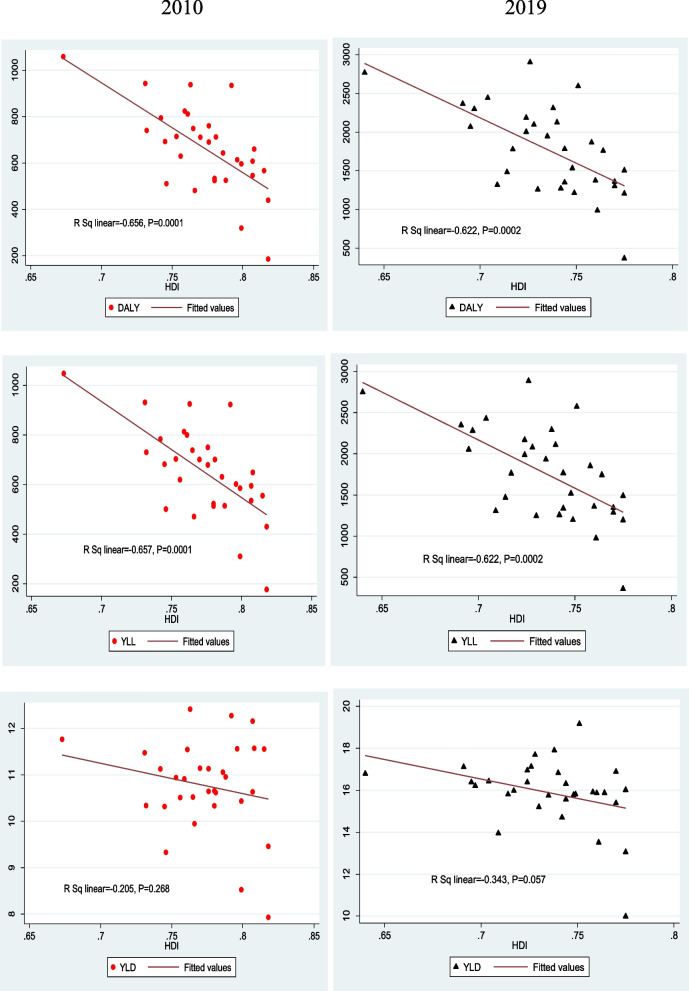
Correlation between the HDI with DALY, YLL and YLD Rates of RTIs in Iran in 2010 and 2019

In regard to the human development index in different provinces of Iran and the burden of road injuries, the finding showed that there was a strong negative correlation between the DALY rate of road injuries with the human development index in 2010 (*r* = −0656, *p*-value = 0.0001) and in 2019 (*r* = −0.622, *p* value = 0.0002). In addition, the findings showed that there was a strong negative correlation between the years of life lost due to premature mortality and HDI in 2010 and 2019. This shows that in provinces with lower human development index, life lost due to premature mortality is higher, the correlation in 2010 and 2019 was (*r* = −0.667, *p*-value = 0.0001) and (*r* = −0.622, *p*-value = 0.0002) respectively. Moreover, the finding showed that there was a negative correlation between the years of life lost due to disability and HDI in 2010 and 2019, but statistically speaking, the correlation wasn’t strong (Fig. 5).

## Discussion

Although children are not allowed to drive motor vehicles, they are vulnerable to road traffic injuries anyway. As children grow up, they have more tendency to go out, and this makes them become more vulnerable to road traffic injuries. Since children are less aware of traffic rules and regulations and they try to cross streets while motor vehicles might be still on the road, this might lead to an accident [21].

Since children are not fully-grown yet and their cognitive development has not been completed, they can’t decide or control a situation, so they are among the most vulnerable people to road traffic injuries [22].

The finding of the study indicated that the incidence of road traffic injuries from 2010 to 2019 in children aged 0–14 in Iran has shown a downward trend. This finding is consistent with the findings of the previous studies that were performed in all age groups in Iran [23–25].

A study conducted by Sadeghian Tafti et al. on the burden of road traffic injuries from 1990 to 2019 indicated that over the years the death rate dropped in all age groups.

There has been a decline in the death rate because of replacing one type of vehicle with another type, (for example replacing a bike with a motorcycle, and replacing a motorcycle with more safe means of transport such as a motor vehicle and public transport) [9]. Taking actions that can reduce the severity of RTIs can cause the mortality rate to drop showing that probably some progress regarding road safety has been made. However, based on studies that have been conducted, the standardized incidence rate and the rate of YLL and YLD per 100,000 persons in Iran are significantly higher than the global rate [26, 27].

This study showed that the mortality rate caused by RTIs in children in Iran has dropped, and for the country to achieve a Sustainable Development Goal (SDG) to reduce 50% of the mortality rate caused by RTIs by 2030, it must invest in resources and infrastructure [7, 24]. Children must be educated on how to stay safe on the roads. Although the current study, as well as the previous ones, show a decrease in road traffic injuries if pedestrians are taught how to be safe on the roads, to keep road traffic injuries decreasing, children need to learn about traffic rules and regulations both in home and school and they should apply what they have learned not only in theory but also in action. What children need to be educated about are as follows: as pedestrians or cyclists they need to learn about road safety, and they should be advised to wear helmets as cyclists and fasten their seatbelt. Other safety measures that children need to be aware of are as follows: they should wear clothes with a bright color so that drivers can see them well, they should wear clothes with bright straps or wear backpacks with bright straps, also they should mount their bike with front and rear light, all these can reduce road traffic injuries in children [22].

It is better to educate children with the above-mentioned training from the beginning of kindergarten (age 5), also, such training must be included in children’s education programs at school [28]. The finding showed that solely 33 percent of children under the age of ten were in the adults’ field of view when a horrific accident happened. Supervising children by adults can reduce road traffic injuries in them. The finding strongly advocate awareness raising about road traffic injuries in children as well as improving parental and caregiver supervision [22, 29].

Installing speed and red-light cameras in areas with a higher risk of RTIs, such as school zone and high-traffic areas would probably prevent RTIs in children.

Based on the study result, the mortality incidence rate in men-children was higher than in female-children. The finding of another study indicated that the burden of road accidents in men is higher compared with women [25, 27].

A study that was performed by (Lee et al., 2018) on children indicated that the rate of RTIs in boys is higher than in girls [28].

This is partly due to the fact that men usually take part in risky behaviors. Such findings indicate how important gender differences are when it comes to developing interventions to prevent road traffic injuries. Based on the need of children, adolescents, and the elderly some age-specific interventions such as designing roads and traffic should be considered.

Meanwhile, children under the age of 15 need educational intervention to learn more tips for travel safety and traffic rules and regulations at school [14, 30, 31]. Vulnerable road users (VRU) are not taken into account while planning, constructing, and designing roads and means of transport in Iran and many low-income countries [32].

The finding of the study indicated that the mortality incidence rate and also the DALY rate for RTIs in children have been significantly different in provinces of Iran. The highest rate of RTIs was related to Sistan and Baluchestan province and the lowest was related to Tehran. The finding of this study is consistent with the finding of the previous study that was conducted by Sadeghian Tafti et al. in all age groups [26]. Similarly, other studies showed a significant difference in the rate of mortality caused by RTIs in all provinces of Iran [32, 33].

The result of the current study indicated that the highest rate of RTIs in boys and girls in 2010 and 2019 were caused by motor vehicle injuries, therefore, knowing the factors that may play an important part in causing injuries is of great importance. In similar studies, parents’ low education, getting involved in accidents in school zones, and playing outside for hours without supervision were considered as the related factors that lead to RTIs in children [29, 34–36].

The finding of the current study indicated that there was a strong negative correlation between the road traffic mortality rate (*r* = −0.613, *p*-value = 0.0002), DALY rate (*r* = −0.622, *p*-value = 0.0002), and the years of life lost from mortality rate (*r* = −0.622, *p*-value = 0.000) with the human development index, and in provinces with lower human development index, the death rate, years of life lost due to premature mortality and DALY are higher. In other countries, inequalities in the burden of road accidents have been also reported [37, 38].

Inequalities might be due to factors such as the geographical scopes of provinces which increase road length and makes it hard to access search and rescue centers. Some other factors are non-standard two-way roads, population structures, vulnerable roadway users, vehicle safety problems, different ways of living, different cultures, gross domestic products, education levels, risky driving behavior, taking drugs, level of economic activities, curvy roads, mountains, rivers, tunnels, and highlands [26]. Some studies have also reported that high population density can lead to lesser road traffic injuries by reducing motor vehicle speed [33, 37].

The ecological design of our study is its primary limitation, as it precludes causal inference and is susceptible to confounding by unmeasured factors. The observed association between higher HDI and lower RTI burden may be influenced by regional variations in factors that are also correlated with development. For example, provinces with a higher HDI likely have better healthcare systems and emergency response capacities, which could reduce case-fatality rates and lead to an underestimation of the true association between socioeconomic factors and injury incidence. Conversely, higher HDI is associated with greater vehicle ownership (motorization) and potentially higher exposure to risk, which, if not fully accounted for, could bias our results. Other factors, such as the quality of road infrastructure, strict enforcement of traffic laws (e.g., seatbelt and helmet use), levels of urbanization, and even geographic terrain, could confound the results presented here. While the use of a composite index like HDI partially captures some of these dimensions, future studies with access to data on these specific confounders at the subnational level are needed to disentangle their individual effects and better understand the mechanisms driving the observed association.

Our study does not predict the likelihood of meeting the SDG target but rather establishes an essential benchmark against which future progress, driven by post-pandemic policies and renewed road safety efforts, can be measured.

Finally, it is required to make some changes in provinces to prioritize evidence-based intervention. Measuring and understanding the burden of road traffic injuries in children will help policymakers and public health planners to make strategic investments to prevent road traffic injuries in children. Performing studies in different provinces and recognizing risk factors could be effective in decreasing mortality and road traffic injury in children.

The GBD methodology provides the only available set of estimates for road traffic injuries (RTIs) that are standardized and comparable across all provinces of Iran and over time. This is paramount for an inequality analysis, as it allows for a direct and unbiased comparison of RTI burden between regions with different socioeconomic statuses (HDI). Other data sources (e.g., national police reports or hospital registries) often suffer from inconsistent definitions, under-reporting, and varying data collection methods, which would severely confound an assessment of true inequalities. GBD provides a complete picture of the health burden beyond just mortality, by generating estimates for incidence, years of life lost (YLL), years lived with disability (YLD), and disability-adjusted life years (DALY). This allows us to explore inequalities in both fatal and non-fatal outcomes, which is essential for a holistic understanding.

## Conclusion

In conclusion, our study provides evidence of a significant decline in road traffic mortality among Iranian children aged 0–14 between 2010 and 2019. Despite this positive trend, road traffic injuries (RTIs) remain a leading cause of death and disability in this age group, underscoring the need for continued focus. Our analysis specifically demonstrates a clear ecological association: provinces with a lower Human Development Index (HDI) consistently exhibited a higher burden of RTIs, evidenced by significantly elevated age-standardized rates of mortality, DALY, YLL, and YLD. Therefore, our results suggest that resource allocation and public health interventions should be prioritized in provinces with lower HDI scores.

## Data Availability

The data used during the present study can be provided by the corresponding author upon reasonable request.
